# Flowers Like α-MoO_3_/CNTs/PANI Nanocomposites as Anode Materials for High-Performance Lithium Storage

**DOI:** 10.3390/molecules28083319

**Published:** 2023-04-08

**Authors:** Laraib Kiran, Mehmet Kadri Aydınol, Awais Ahmad, Syed Sakhawat Shah, Doruk Bahtiyar, Muhammad Imran Shahzad, Sayed M. Eldin, Aboud Ahmed Awadh Bahajjaj

**Affiliations:** 1 Chemistry Department, Quaid-i-Azam University, Islamabad 45320, Pakistan; lkiran@chem.qau.edu.pk; 2 Nanosciences and Technology Department (NS&TD), National Centre for Physics (NCP), Islamabad 44000, Pakistan; 3 Metallurgical & Materials Engineering Department, Middle East Technical University, Ankara 06800, Turkey; kadri@metu.edu.tr (M.K.A.); doruk.bahtiyar@metu.edu.tr (D.B.); 4 ENDAM, Energy Materials and Storage Devices Research Center, Middle East Technical University, Ankara 06800, Turkey; 5 Department of Chemistry, University of Lahore, Lahore 54000, Pakistan; 6 Departamento de Quimica Organica, Universidad de Cordoba, 14014 Cordoba, Spain; 7 Faculty of Engineering and Technology, Future University in Egypt, New Cairo 11835, Egypt; elsayed.research@fue.edu.eg; 8 Department of Chemistry, College of Science, King Saud University, Riyadh 11451, Saudi Arabia; mmetwallyahmedsyed@gmail.com

**Keywords:** α-MoO_3_, CNTs, PANI, anode materials, lithium-ion battery

## Abstract

Lithium-ion batteries (LIBs) have been explored to meet the current energy demands; however, the development of satisfactory anode materials is a bottleneck for the enhancement of the electrochemical performance of LIBs. Molybdenum trioxide (MoO_3_) is a promising anode material for lithium-ion batteries due to its high theoretical capacity of 1117 mAhg^−1^ along with low toxicity and cost; however, it suffers from low conductivity and volume expansion, which limits its implementation as the anode. These problems can be overcome by adopting several strategies such as carbon nanomaterial incorporation and polyaniline (PANI) coating. Co-precipitation method was used to synthesize α-MoO_3_, and multi-walled CNTs (MWCNTs) were introduced into the active material. Moreover, these materials were uniformly coated with PANI using in situ chemical polymerization. The electrochemical performance was evaluated by galvanostatic charge/discharge, cyclic voltammetry (CV) and electrochemical impedance spectroscopy (EIS). XRD analysis revealed the presence of orthorhombic crystal phase in all the synthesized samples. MWCNTs enhanced the conductivity of the active material, reduced volume changes and increased contact area. MoO_3_-(CNT)_12%_ exhibited high discharge capacities of 1382 mAhg^−1^ and 961 mAhg^−1^ at current densities of 50 mAg^−1^ and 100 mAg^−1^, respectively. Moreover, PANI coating enhanced cyclic stability, prevented side reactions and increased electronic/ionic transport. The good capacities due to MWCNT_S_ and the good cyclic stability due to PANI make these materials appropriate for application as the anode in LIBs.

## 1. Introduction

The current fossil fuels-based energy is at a severe risk owing to many factors, including the consumption of non-renewable energy resources. Another worrying aspect of the current fossil fuel energy economy is connected with CO_2_ emissions, which has increased at a uniform rate, thus resulting in an increased global temperature with a series of sudden climate changes. Environmental pollution and the growing population have caused an energy crisis, therefore it is very difficult to meet the current energy demands. Consequently, researchers are trying to develop affordable, ecofriendly and environment-friendly energy storage devices using low cost, potentially abundant and environment-friendly material. The development of renewable energy technologies is a substantial approach to limit global warming, environmental pollution and the deficiency of fossil-based resources. Energy storage plays a vital role in developing renewable energy systems [1,2,3,4]. Electrochemical systems such as super capacitors and batteries, which can effectively deliver and store energy in power plants, can also provide load levelling and power quality in the integrated systems [5,6].

In this context, lithium-ion batteries (LIBs) have been proved as promising energy storage devices that are widely used in daily life, such as in hybrid electric vehicles, space exploration, aviation [7] and portable electronic devices [8,9,10], owing to high power densities, environmental friendliness, long cycle lives, low self-discharges, high energy densities, small ionic sizes (which permit fast Li^+^ intercalation in solids) that are a main factor for fast charging, cyclic stabilities, small memory effects and high open circuit voltages [11,12]. To fulfill the current energy demands, the electrochemical performance of LIBs including cycle life, capacity, power density and charging speed should be enhanced [7]. Since the mechanism of LIBs is based on the movement of Li^+^ ions between the anode and cathode, the electrochemical and physical properties of the electrode materials have significant influence on the performance of the battery; typically, a variety of lithium metal oxides such as LiCoO_2_ have been used as the cathode material [12], and graphite is mainly used as the anode material in commercial LIBs owing to low cost, stable operational potential and environmental friendliness [13]. However, the graphite anode exhibits poor rate performance owing to a low theoretical capacity of 327 mAhg^−1^ [10]; it also suffers from lithium dendrite formation, slow ion diffusion coefficient and volume deformation [13]. Moreover, a large hysteresis between lithiation/delithiation causes difficulties in practice applications. Therefore, there is a strong motivation to develop some novel anode materials (an important component of LIBs) with long cycle lives, large capacities and excellent capacity retention [14]. To substitute graphite, transition metal oxide (TMO) anodes, a class of inorganic materials, have been extensively used because of its high theoretical capacity [15,16]. Thus, many transition metal oxides such as Co_3_O_4_, CuO, MoO_3_, WO_3_, NiO and SnO_2_ have been prepared. Moreover, Si nanostructures, metal sulfides [17] and tin compounds have also been developed in the field of lithium-ion battery owing to their large theoretical capacities, widespread availability and environmental friendliness [7,18]. Chu et al. prepared MFe_2_O_4_@HPSs particles confined with a carbon network that exhibited high cyclic stability and good rate performance. Moreover, these nanoparticles maintain structural integrity during charging and discharging [19]. Qinglin et al. prepared ZnO/ZnFeO_4_ nanospheres. Impressively, ZnO/ZnFeO_4_ showed good cycling performance (1137 mAhg^−1^ after 80 cycles at 1 Ag^−1^) [20]. Among all of them, transition metal oxides possess variable valence states and diverse morphology [17]. The capacity of a TMO can reach values of 700–1200 mAhg^−1^. Molybdenum-based materials have been proved as promising electrodes for energy storage systems owing to low cost, multiple valence states and high theoretical capacity [21]. Molybdenum oxide electrodes have been employed as negative and positive electrode materials. Among all of molybdenum oxides, MoO_2_ and MoO_3_ are mostly used [21]. To date, MoO_3_ is used as a promising anode material for Li ion batteries (LIBs) owing to versatile structure, low cost, nontoxicity, natural abundance, adjustable chemical state, high thermal and chemical stability [8], environmental friendliness and a good theoretical capacity of 1117 mAh/g [8], which is three times greater than that of commercial graphite (327 mAh/g) [22]. On the basis of crystallographic arrangement, MoO_3_ has three different polymorphs, h-MoO_3_ (hexagonal), metastable [21] β-MoO_3_ (monoclinic) and α-MoO_3_ (orthorhombic) [7,23], of which α-MoO_3_ is the most stable thermodynamically [7,24]. Moreover, MoO_6_ is a building block of MoO_3_, and MoO_6_ octahedron in the layered structure of MoO_3_ provides a diffusion path for Li ions [23,25,26]. However, α-MoO_3_ exhibits poor electronic and ionic conductivity and suffers from large volume changes that induce pulverization during delithiation/lithiation, unstable crystal structure, small surface area [21], resulting low specific capacity and poor cycling stability [27,28,29]. During cycling, a major issue with transition metal oxides is pulverization and cracking, which causes aggregation and dispersion in electrode material [12]. In order to cope with these kinds of problems, multiple strategies have been applied to improve electrochemical performance, mechanical strength, surface area, electron and mass transport kinetics and conductivity of TMOs, such as designing nanostructures, introducing conductive agents and engineering defects [21]. MoO_3_ has been converted into various forms such as nanowires, nanorods, nanobelts [30], nanofibers and nano sheets [24]. The small size of nanoparticles shortens Li^+^ diffusion path and the large surface area provides active sites [31]. Furthermore, one of the most important approaches to increase electronic conductivity of α-MoO_3_ and to improve Li^+^ ion diffusion and electronic conductivity, carbon materials and matrices have been introduced into MoO_3_ such as carbon nanotubes (CNTs), carbon black (graphene) [32] and polyaniline (PANI).

Moreover, the conducting polymer also provides the conducting backbone, which increases the lithium ion conductivity, stability [33] and electrochemical performance of the electrode [34,35].

Polymer materials such as polypyrrole (Ppy) and polyaniline (PANI) [24] have been coated and the performance has improved. Recently, nanocomposites of MoO_3_ with poly-pyrrole were synthesized and the nanocomposites exhibited good cycling stability and electrochemical performance in lithium ion batteries, signifying the successful use of the polymer [36]. Polyaniline is considered another polymer for a potential conductive polymer due to its environmental stability, easy synthesis and high conductivity [37]. PANI has a function of improving conductivity and stabilizing the structure. Cai et al. utilized nest-like PANI that reduced volume changes to increase electrochemical performance of Si-based anode materials [37]. Wu et al. prepared a 3D hydrogel conducting network by in situ polymerization that demonstrated good cycling performance [38]. Furthermore, PANI coating on the surface of MoO_3_ acts as a good anode material for a Li^+^ ion host.

Moreover, carbon also acts as a buffer that reduces the stress owing to a large volume of expansion during the charging–discharging by enhancing the electrical conductivity of anodes [39,40,41]. Furthermore, carbon increases the structural stability by surrounding the active material particles because it prevents active material aggregation during cycling. However, comparatively, CNTs displayed a good rate capability and cyclic stability over carbon [42].

Conductive carbonaceous materials such as CNTs and reduced graphene oxide (RGO) have been used with MoO_3_, which can improve conductivity and structural stability, thus increasing the overall electrochemical performance [43]. The unique properties of CNTs such as low density and high conductivity make them well suited to synthesize TMO–CNT nanocomposites for the lithium ion battery. Therefore, we developed a straightforward and facile co-precipitation method for the synthesis of MoO_3_, MoO_3_-PANI, MoO_3_-(CNTs)_x%_ and MoO_3-_(CNTs)_x%_-PANI. The battery parameters, such as coulombic efficiency, cyclic stability and capacity retention are compared and discussed with MoO_3_ bulk.

## 2. Results and Discussion

**Figure 1:**
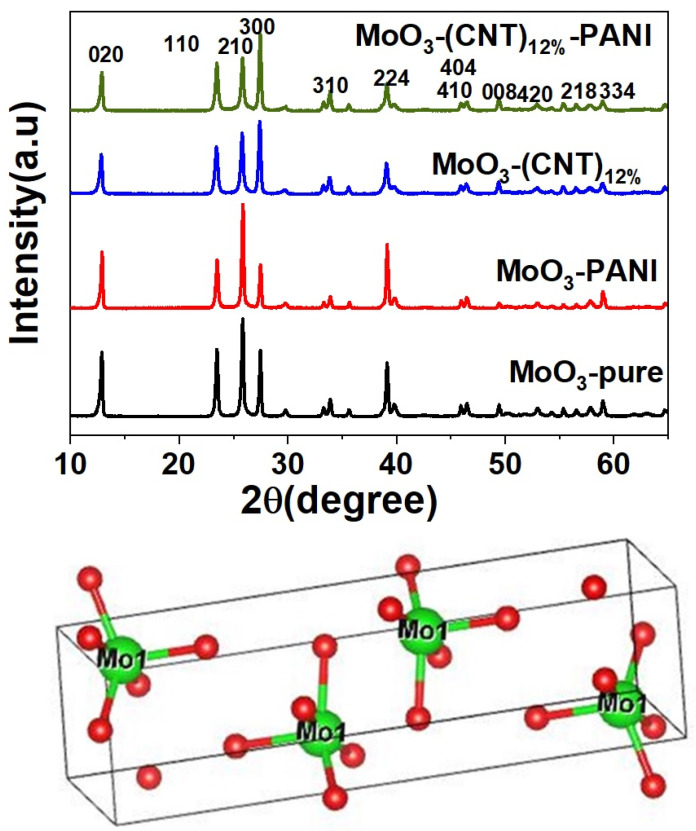
XRD patterns of samples and the orthorhombic crystal structure of MoO_3_.

Figure 1 depicts XRD patterns for MoO_3_-pure and its nanocomposites, indicating orthorhombic MoO_3_ phase for all samples with no diffraction peaks of other impurities with the lattice parameters (a = 3.962 Å, b = 13.85 Å, c =3.6970), which are well consistent with the JCPDS card no 05-058 [44,45,46,47]. The three sharp peaks at 23.46, 25.82 and 27.46 can be perfectly indexed to the crystalline orthorhombic phase; however, other minor peaks also showed a best match with the JCPDS card [48]. This observation shows that PANI coating does not affect the crystal lattice of MoO_3_ and that the structure is preserved. The crystallite sizes for various synthesized samples were calculated using Debye–Scherrer equation as represented: λ is incident wavelength of X-rays, which is 1.5406 nm, β is the full width at half-maximum of the selected peak in radians and θ is the diffraction angle (Bragg’s angle) at which the peak arises and is also measured in radians [49]. The crystallite sizes of the materials are as follows: MoO_3_ (48.6 nm), MoO_3_-PANI (41.43 nm), MoO_3_-(CNT)_12%_ (39.7 nm) and MoO_3_-(CNT)_12%_-PANI (47 nm). All information regarding other samples are given in Table S1 (supporting information) and Figure S1.

D = kλ/βcosθ(1)

where D represents crystallite size and k represents the constant usually taken as 0.9. The crystal structure of α-MoO_3_ is shown at the bottom of Figure 1, in which the red balls represent oxygen and the green balls indicate molybdenum (Mo). 

**Figure 2:**
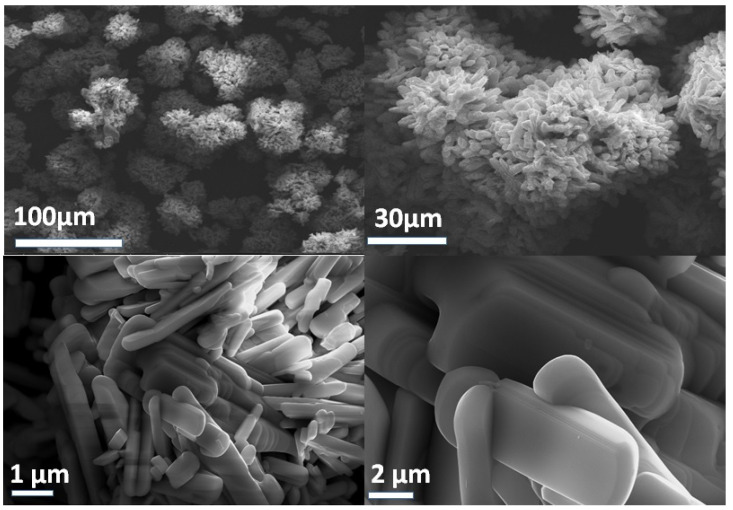
FESEM images of MoO_3_-pure.

The surface morphologies of the MoO_3_-pure and their nanocomposites were investigated by SEM. Pure α-MoO_3_ presented with a uniform plate-like structure that appears like clusters of nanoflowers at low magnification, as shown in Figure 2. At high magnification, it showed a flat smooth surface with a semicircular tip. These plates [50] combined layer by layer to form well oriented microbelts, showing perfect morphology and well crystallinity.

**Figure 3:**
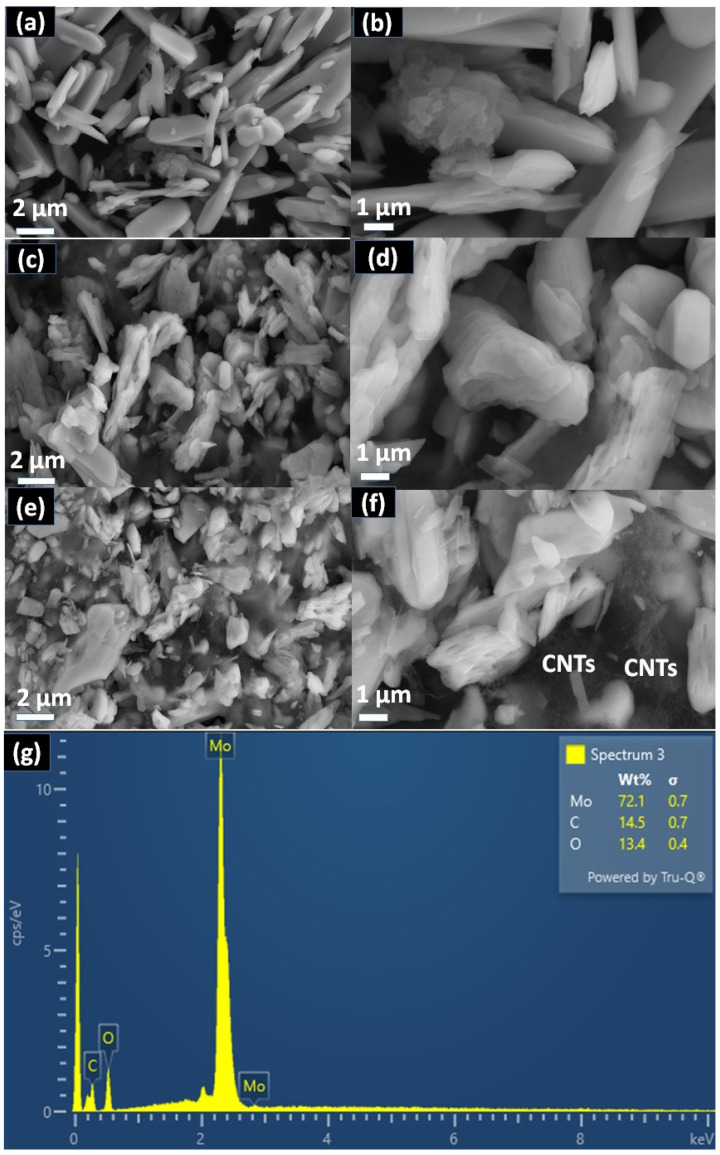
FESEM images of samples (**a**,**b**) MoO_3-_PANI, (**c**,**d**) MoO_3_-(CNT)_12%_ and (**e**,**f**) MoO_3_-(CNT)_12%_-PANI and (**g**) EDX spectrum of MoO_3_.

MoO_3_ maintained a plate-like morphology after the addition of CNTs, but with a small size, unsmooth particle surface and a little disorder, as shown in Figure 3. It was also observed that CNTs were well dispersed around the MoO_3_ nanoparticles. EDX analysis was also done to determine relative abundance and elemental composition. The corresponding EDS mapping exhibited existence of Mo, O and C.

**Figure 4:**
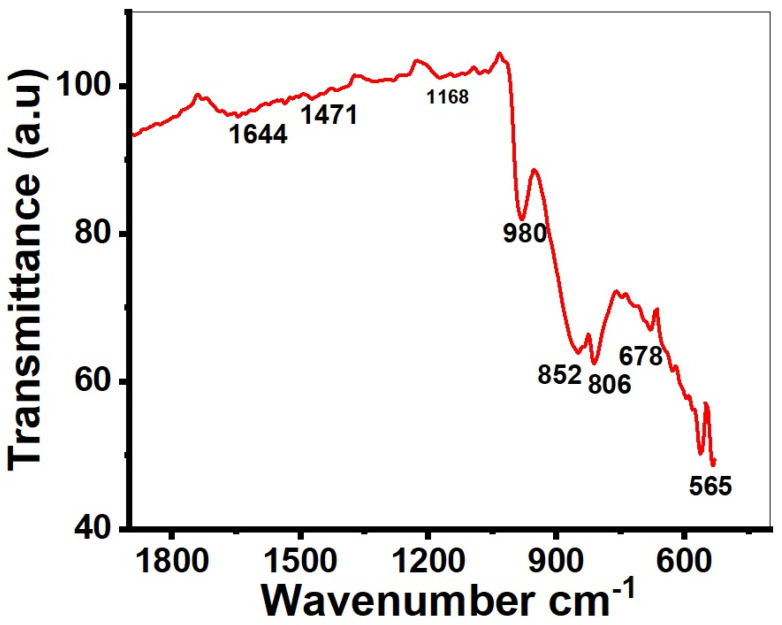
FTIR spectrum of the calcined MoO_3_.

FTIR (Fourier transform infrared) spectroscopy was employed to investigate the bonding and functional groups of MoO_3_. FTIR analysis was carried out in the transmittance mode for structure elucidation as well as to assess bonding present between various components, as shown in Figure 4. Two peaks were observed at 565 cm^−1^ and 678 cm^−1^, displaying the bending and stretching vibration of MoO [51]. The peaks at 852 cm^−1^ and 980 cm^−1^ are assigned to the stretching vibration of the O–Mo–O bond and the stretching vibration of oxygen in Mo–O–Mo, repectively [52]. The small peaks observed at 1471 cm^−1^ and 1644 cm^−1^ correspond to the bending vibrations of H–O–H [53]. It also showed the presence of water in crystallization of MoO_3_.

**Figure 5:**
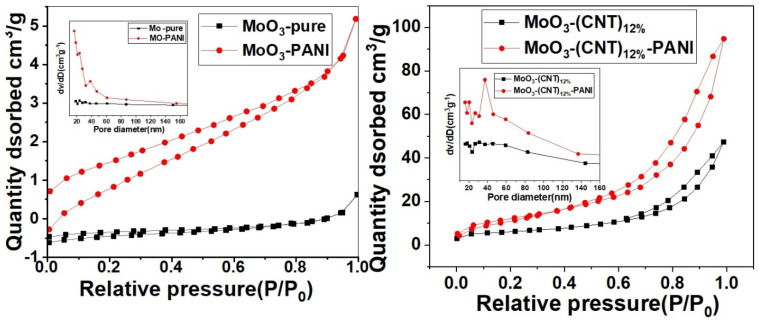
N_2_ adsorption/desorption isotherm of MoO_3_ at 77 K and pore size distribution curve in the inset of the figure.

Pore-size distributions and the nitrogen adsorption–desorption isotherms of MoO_3_-pure, MoO_3_-PANI, MoO_3_-(CNT)_12%_ and MoO_3_-(CNT)_12%_-PANI composites are shown in Figure 5. MoO_3_ showed almost no adsorption at a relative pressure less than 0.9, while it started showing small adsorption when relative pressure approached 1.0. On the contrary, in the case of MoO_3_-CNTs_(12%)_-PANI, with the increase of relative pressure, adsorption quantity increased gradually. In addition, in the 0.9–1.0 relative pressure range, hysteresis loop was observed. The BET surface areas of MoO_3_, MoO_3_-PANI, MoO_3_-(CNT)_12%_ and MoO_3_-(CNT)_12%_-PANI were 0.849 m^2^/g, 4.017 m^2^ g^−1^, 19.8 m^2^ g^−1^ and 41.3 m^2^ g^−1^, respectively, and the BJH (Barrett-Joyner-Halenda) pore volumes were 0.0018 cm^3^g^−1^, 0.008 cm^3^g^−1^, 0.072 cm^3^g^−1^ and 0.143 cm^3^g^−1^, respectively. MoO_3_-(CNTs)_12%_-PANI displayed a broad pore-size distribution in the 5–150 nm pore diameter range. The porous structure with a large surface area makes charge transfer easier at the electrode/electrolyte interface due to additional access points to the electrolyte.

**Figure 6:**
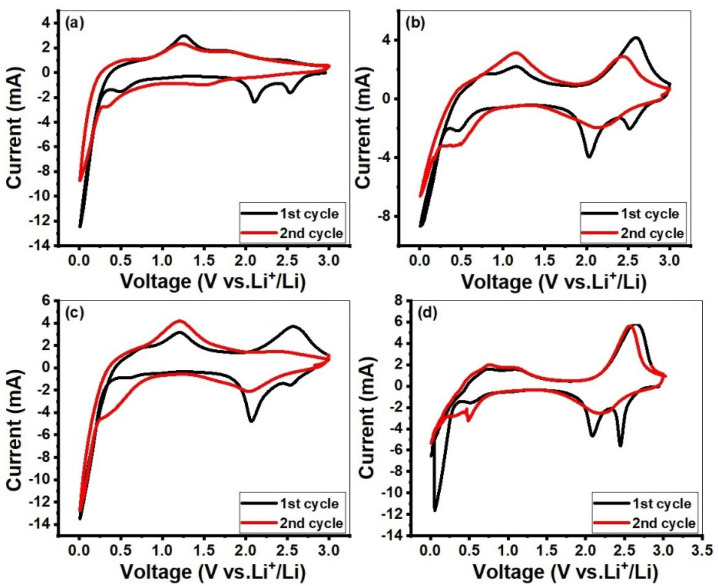
Cyclic voltammogram curves of all nanocomposites: (**a**) MoO_3_, (**b**) MoO_3_-PANI, (**c**) MoO_3_-CNT_S(12%)_ and (**d**) MoO_3_-CNTs_(12%)_-PANI nanocomposite.

Figure 6 shows the CVs of all the MoO_3_-based electrodes at the scan rate of 0.5 mVs^−1^ over a potential range of 0.01–3.0 V for the first two cycles. Three broad peaks were seen at 2.11 V, 2.57 V and 0.01 V vs. Li^+^/Li in the first cycle of the cathodic polarization process, which corresponds to the lithium intercalation processes, while the current anodic peak at 1.25 V was seen in anodic polarization, which corresponds to the lithium extraction process. More specifically, these types of anodic and cathodic peaks at these potentials represent lithium insertion/de-insertion in different structural sites of MoO_3_ to form Li_x_MoO_3_. The strong peak at 0.01 V appeared due to lithiation of MoO_3_. The area of peak in the first cycle was more than that in the second cycle, which may be attributed to irreversible conversion reactions [54]. The change in the shape of the cathodic peak in the second cycle corresponds to the structural change of the material. When the lithium cation enters into the structure, it increases interlayer spacing and the repulsive forces of the cation leads to cracking of MoO_3_ and thus decreases the particle size. That is why the peaks of MoO_3_ change in the next cycles [55,56]. The overall discharge and charge process could be summarized by the Equations (1) and (2) [57]:

Intercalation: MoO_3_ + xLi^+^ + xe^−^   →   Li_x_MoO_3_ (2)

Conversion:    Li_x_MoO_3_ + (6 − x) Li^+^ + (6 − x) e^−^   ↔    Mo + 3Li_2_O (3)

**Figure 7:**
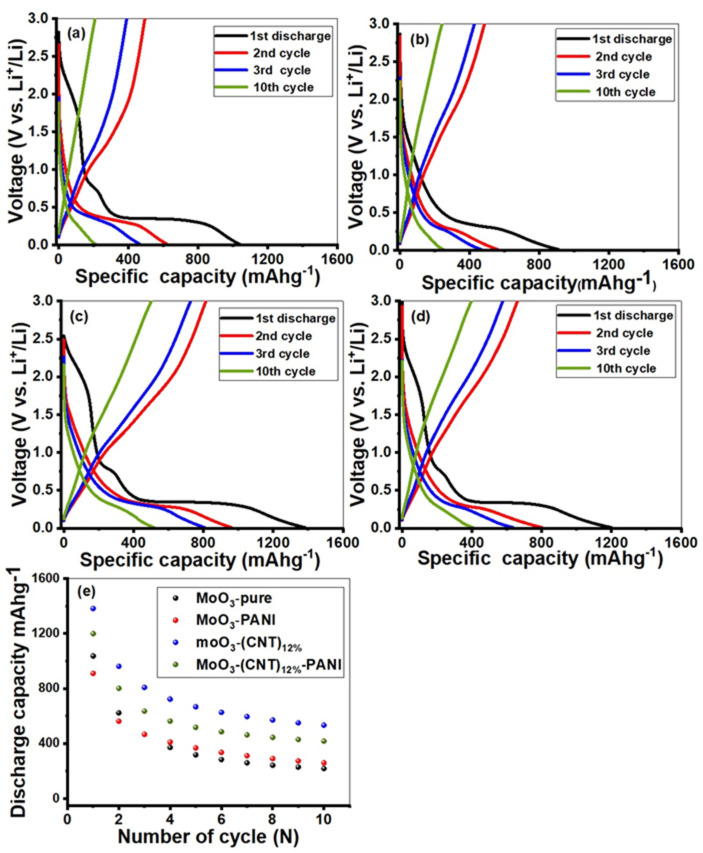
The charge/discharge curves of (**a**) MoO_3_, (**b**) MoO_3_-PANI, (**c**) MoO_3_-(CNT_S_)_12%_ and (**d**) MoO_3_-(CNTs)_12%_-PANI nanocomposite and (**e**) the cyclic performance of all nanocomposites electrodes in the initial 10 cycles as a function of capacity.

Galvanostatic charge/discharge measurements were performed between the voltage windows of 0.01–3 V at a current density of 100 mAg^−1^, as shown in Figure 7. The initial discharge capacities of MoO_3_-pure, MoO_3_ PANI, MoO_3_-(CNT)_12%_ and MoO_3_-(CNT)_12%_-PANI at 100 mAhg^−1^ were found to be 622.23, 561.75, 961.50 and 801.00 mAhg^−1^, respectively, while the charge capacities were 494.08, 485.01, 814.04 and 801.00 mAhg^−1^, respectively and the first coulombic efficiencies were 79.40, 86.30, 84.60 and 82.40%, respectively. All MoO_3_ anodes led to low coulombic efficiencies in the first cycle, because of large irreversible capacities [55]. The large irreversible capacity arises owing to the following reasons: firstly, the solid electrolyte interface (SEI) formation on the surface of nanoparticles; secondly, decomposition of electrolytes owing to unsaturated carbon atoms. In addition, Li ions may be trapped in the cavities of nanocomposites due to the slow release of Li kinetics, formation of lithium compounds or may be due to the bonding between less coordinated atoms at defect sites [55,58]. From the second discharge onwards, the discharge/charge curves were well coincided. All uncoated samples almost showed similar behavior, showing variations only in the extent to which these materials stored capacity and further how much capacity was retained upon cycling. The first discharge profile showed a rapid potential drop from 3 V until it reached a narrow plateau around 2.3 V, which is due to the intercalation of lithium ions into the crystal structure of the active material, followed by a wide plateau at a potential of 0.8 V, which corresponds to the conversion reaction between lithium ions and molybdenum oxide. Moreover, a steep profile followed this narrow plateau, which tappers off gradually until the potential reached 0.01 V and showed the formation of SEI. The following second discharge curve underwent changes due to the Li^+^ driven structural changes and therefore did not show any narrow plateau at a potential lower than 2.3 V, followed by a reduction in the wide plateau and steep profile. Information regarding other compositions of α-MoO_3_ are given in supporting information (Figure S3).

**Figure 8:**
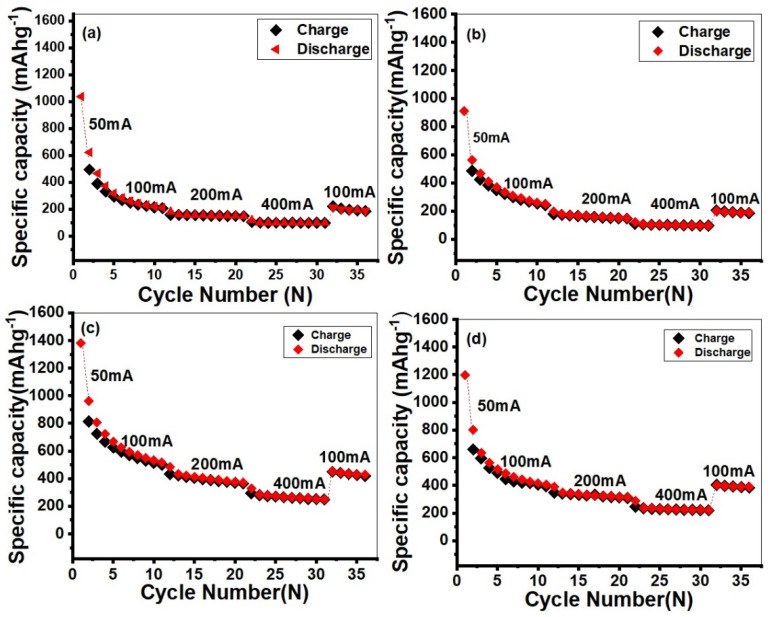
The cyclic performances of (**a**) MoO_3_, (**b**) MoO_3_-PANI, (**c**) MoO_3_-(CNT_S_)_12%_ and (**d**) MoO_3_-(CNTs)_12%_-PANI nanocomposites at various current rates.

Figure 8 investigates specific capacity, rate performance and cyclic stability of MoO_3_-pure, MoO_3_-(CNT)_x%_ and MoO_3_-(CNT)_x%_-PANI electrodes at various rates, such as 50 mAg^−1^, 100 mAg^−1^, 200 mAg^−1^ and 400 mAg^−1^ for 35 cycles. The charge/discharge capacities of Mo-(CNT)_12%_ were 814.5/961.5, 433.8/484.9 and 293.9/330.0 and of MoO_3_-CNT)_12%_-PANI nanocomposites were 660.0/801.0, 347.0/390.7, 247.5/286.9 mAg^−1^ at the current density of 100, 200 and 400 mAg^−1^, respectively; while pure MoO_3_ possessed charge/discharge capacities of 494.08/622.23, 160.20/181.40 and 104.10/119.70 at the same current rates, respectively. The graph shows a decrease in capacity with an increase of current density; however, when the current density reduced back to 100 mAg^−1^, a charge/discharge capacity of 449.93/450.96 and 211/221 in the cases of MoO_3_-(CNT)_12%_ and pure MoO_3_, respectively, were obtained and remained stable in the subsequent cycles. PANI occupied some spaces in the nanoparticles, which increased reversibility and cyclic stability owing to the increasing extraction/insertion of the Li^+^ ion. Carbon nanotubes also improved the performance of the composites because of their conductivity; they also prevented the electrode from disintegrating during charge and discharge. Apart from these, carbon nanotubes were active electrochemically and prevented electrode disintegration by providing a mechanical framework during charge and discharge [59]. On the other hand, carbon nanotubes provided a physical barrier to prevent MoO_3_ nanoparticles from aggregation and improved structure integrity [60]. Moreover, during cycling, they acted as buffers that accommodate volume changes and thereby maintained structural stability [60]. Specific capacity decreased drastically by increasing the cycle number due to volumetric changes that resulted in a decrease in crystallinity. The stability results of MoO_3_-(CNT)_12%_ were better compared to those of MoO_3_-(CNT)_12%_-PANI (Table 1).

**Figure 9:**
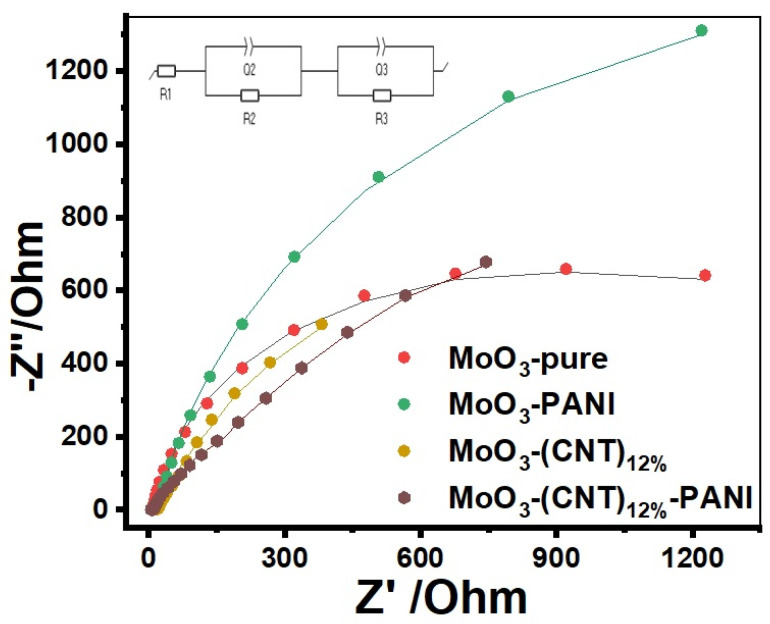
Electrochemical impedance spectra of MoO_3_ electrodes, symbols (experimental data), and solid line (simulated data) at open circuit potential (OCP).

The Nyquist plots of the EIS spectra Z” (ohm) and Z’ (ohm) represent imaginary and real impedance components in the Nyquist plots. To evaluate the kinetics of anode and cathode electrodes and to provide information about the bulk resistance of the electrode, surface film and charge transfer, we measured the electrochemical impedance spectroscopy (EIS) of the MoO_3_-pure, MoO_3_-PANI, MoO_3_-(CNT)_12%_ and MoO_3_-(CNT)_12%_-PANI nanocomposite electrodes. An equivalent circuit was employed to model the circuit parameters corresponding to the EIS of the cell. The electrochemical impedance spectra (EIS) of nanocomposites were recorded at an open circuit potential (OCP) as shown in Figure 9. R1 connected in series represents the solution resistance, and the two parallel circuits consisting of constant phase elements (CPEs) and resistance (R) represent the capacitive and resistive load in the cell. R_2_ and CPE_2_ denote the resistance and constant phase element of the semicircle obtained at the high frequency region, while R_3_ and CPE3 are for the semicircle obtained at the low frequency region. According to the literature, the small semicircle in the high frequency region signifies interfacial layers, where Li^+^ ions migrate through surface films on the electrode encounter resistance. The larger semicircle represents charge transfer resistance and double layer capacitance at the low frequency range. The semicircles, however, are generally depressed and a true capacitor cannot fit well with the experimental data. The CPEs are generally used in place of a capacitor to obtain a better mathematical fit, but their physical justification is not obvious. Heterogeneities in the electrodes, such as surface roughness and porosity, were generally attributed with this observation [63,64]. Physical interpretation of Q and α values in CPE is not straightforward and has no clear physical correspondence. Only alpha (α) can provide a measure of how similar the CPE is to an ideal capacitor. In addition, alpha being close to 0.5 was attributed to the behavior of a porous electrode, whereas for an ideal flat electrode, it is unity. Beyond these, Q and α hardly provide any physical insight and were not analyzed in detail [65].The experimental data (symbols) and the simulated (solid line) data according to the electrical equivalent circuit in the inset of figure are collected in Table 2. The chi-square (χ2) showed an acceptable correlation between simulated and experimental data, thereby validating the equivalent circuit model. At the electrode–electrolyte boundary, there was kinetic resistance offered owing to the charge transfer [66]. MoO_3_-(CNT)_12%_ showed small resistance due to the electric conductivity offered by CNTs and the small contact resistance of the active material and current collector [67]. 

## 3. Materials and Methods

### 3.1. Materials

All chemicals and reagents were purchased from Sigma-Aldrich (Saint Louis, MO, USA) and used without any further purifications.

### 3.2. Synthesis of α-MoO_3_

MoO_3_ nanorods were synthesized by adding 0.1 M solution of ammonium heptamolybdate tetrahydrate in 100 mL deionized water, and the solution was stirred continuously for 30 min. Subsequently, 5 mL concentrated nitric acid (HNO_3_) was poured slowly drop-wise. Pale-yellow colored precipitates were observed in a reaction beaker. The beaker was then placed in a water bath at 120 °C for 3 h. The obtained precipitates were washed, centrifuged and dried for 6 h in an oven at 70 °C.

### 3.3. Synthesis of α-MoO_3_-MWCNTs-PANI

**Figure 10:**
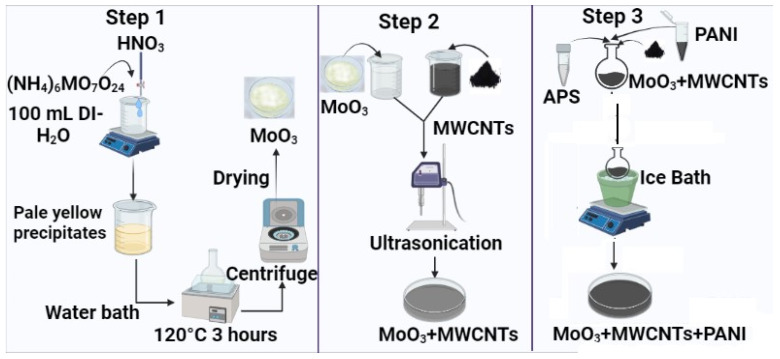
Schematic diagram of the formation of MoO_3_-(CNT)_x%_-PANI composites.

Accordingly, for the preparation of MoO_3_/MWCNTs, (x = 4% (0.15 g), x = 8% (0.312 g), x = 12% (0.48 g)) of functionalized MWCNT_S_ were ultrasonicated for 30 min in 50 mL water (solution A). A quantity of 3.62 g of prepared MoO_3_ was also ultrasonicated for 30 min in 25 mL water. A total of 25 mL of solution B was transferred to 50 mL of solution A, and the mixture was ultrasonicated for one hour. Excess water was removed using a centrifuge and the remaining was dried in an oven at 50 °C. For the preparation of MoO_3_/MWCNTs/PANI, an appropriate amount of prepared material was added in a round bottom flask containing 1 M HCl solution and ultrasonicated for half hour, as shown in Figure 10. The solution was then placed in an ice bath, so that during the whole reaction time, the temperature could be maintained. After 10 min of stirring, 10 wt% of aniline monomer was added in a round bottom flask, followed by 30 min of stirring. A calculated amount of ammonium per sulfate (APS) was added in a small amount of distilled water and poured slowly in a round bottom flask. APS acts as an oxidizing agent that mainly initiates polymerization of aniline monomers. The reaction mixture was stirred in an ice bath for 6 h and the temperature was maintained below 4 °C. After 6 h, the mixture was washed many times and dried.

### 3.4. Physiochemical Characterization

The crystal structure of the sample was investigated by XRD (X-ray diffraction) with Cu kα radiation (λ = 0.154 nm), recorded by Bruker D8 diffractometer. FESEM (field emission scanning electron microscopy) (FEI 430 Nano Scanning Electron Microscope, Ankara, Turkey) was used to confirm the surface morphology. EDX (energy-dispersive X-ray spectroscopy, Turkey) was used for elemental mapping. The pore size distribution and specific surface areas were analyzed by N_2_ adsorption–desorption isotherm. FTIR (JASCU 6600, NCP, Islamabad, Pakistan) analysis was done to identify the functional groups of MoO_3_.

### 3.5. Electrochemical Measurements

Electrochemical measurements were done by using two electrodes and a lithium foil as a reference or counter electrode. The electrode was fabricated by mixing active material, PVDF binder and conductive carbon black in N-methyl-2-pyrrolidinone (NMP) in a weight ratio of (75:15:10). By using the doctor blade, the slurry was evenly pasted on a Cu foil (thickness: 200 μm) and then heated overnight in a vacuum oven. The assembling process was completed in a glovebox under argon (Ar) atmosphere, where concentration of O_2_ and H_2_O were below 0.1 ppm. The discharge/charge measurements were done in the voltage range of 0.00–3.0 V at different current densities. CV measurements were performed at a scan rate of 0.5 mVs^−1^. EIS (electrochemical impedance spectroscopy) was performed at room temperature between the frequency range of 100 kHz and 10 mH_Z_ and an amplitude of 10 mV.

## 4. Conclusions

The increasing global energy crisis and environmental concerns have stimulated the development of energy storage devices that are efficient and clean for the society. Currently, batteries and SCs have exhibited their potential as energy storage devices, as a consequence of excellent charge–discharge capabilities, their great energy densities and long cycling stabilities. MoO_3_ electrodes underwent fast growth and their potential in advancement of effective energy storage systems is explained due to their excellent electrochemical performances and good physicochemical properties. However, their low electrical conductivity has retarded their good energy storage application. As a result, to solve this problem, many nanostructured MoO_3_ and their composites were fabricated. MoO_3_ and their composites with CNTs and PANI coating proved to have excellent electrochemical performances in batteries and SCs. In this work, MoO_3_, MoO_3_-(CNT)_x%_ and MoO_3_-(CNT)_x%_-PANI composites with a plate-like morphology, in which MWCNTs were anchored, were synthesized via the co-precipitation method. Various conditions including temperature and acid concentration were optimized to achieve the desired orthorhombic crystal phase. Moreover, these materials were uniformly coated with PANI by in situ chemical polymerization. The morphology and structure of nanocomposites were investigated by scanning electron microscopy (SEM) and X-ray diffraction (XRD), while the specific surface area and porosity were determined using Brunauer-Emmett-Teller (BET) and Barrett-Joyner-Halenda (BJH) methods. Fourier transform-infrared (FTIR) spectrometer analysis was performed in the transmittance mode in the range of 500 to 4000 cm^−1^ to investigate different functional groups. The electrochemical performance was evaluated by galvanostatic charge/discharge, cyclic voltammetry (CV) and electrochemical impedance spectroscopy (EIS). Pure α-MoO_3_ and its composites with CNT_S_ were uniformly coated with polyaniline by applying in situ chemical polymerization. The PANI coating layer alleviated volume changes and improved conductivity and cyclic stability. MoO_3_ with CNTs contributed to fast Li ion diffusion and buffered the volume changes during this work, which further demonstrates the effect of PANI coating on the LiB performance. The CNTs and PANI suppressed aggregation to maintain structural integrity and improved kinetic and conductivity of MoO_3_. The result showed an increase in specific capacities, particularly in the case of CNT-based composites, where the capacity is recorded as 1382 mAhg^−1^ at 50 mAhg^−1^ and 961 at 100 mAhg^−1^ in the case of MoO_3_-(CNT)_12%_. In short, this study suggests strategies of CNT incorporation and polymer coating to address the problems of low electronic and ionic conductivity and the swelling of active materials during cycling that were successful, and in the future, these can be applied to other active materials as well along with changing the morphology from micro to nanoscale.

## Figures and Tables

**Table 1:** Specific capacities and cycle performances of MoO_3_-based anodes in LIBs as reported in the literature.

Electrode Material	Initial Cycle Discharge (mAhg^−1^)	Reversible Capacity (mAhg^−1^)	Current Density (mAg^−1^)	Ref.
MoO_3_-(CNT)_12%_-PANI	801	406	100	This Work
MoO_3_-(CNT)_12%_	961	517	100	This work
α-MoO_3_	301	180	30	[61]
α-MoO_3_	211	133	300	[61]
MoO_3_	668	157	200	[52]
MoO_3_	974	286	100	[62]
MoO_3_-NiMoO_4_	1031	324	100	[62]
α-MoO_3_-CNT	583	194	500	[8]

**Table 2:** EIS fitting results of samples.

No	Samples	R_1_ (Ω)	R_2_ (Ω)	Q_2_ (Fs^α−1^)	α_2_	R_3_ (Ω)	Q_3_ (Fs^α−1^)	α_3_	χ^2^
1	MoO_3_	7.11	9.92	1.21 × 10^−3^	0.474	1208	4.39 × 10^−3^	0.904	0.0077
2	MoO_3_ PANI	2.66	20.01	0.16 × 10^−3^	0.644	1201	5.25 × 10^−3^	0.882	0.00279
3	MoO_3_ (CNT)_12%_	3.07	22.20	0.11 × 10^−3^	0.678	339	0.0133	0.788	0.00796
4	MoO_3_-(CNT)_12%_ PANI	5.16	45.40	7.65 × 10^−5^	0.722	696	4.64 × 10^−3^	0.754	0.00383

## Data Availability

Not applicable.

## Supplementary Materials

The following supporting information can be downloaded at: https://www.mdpi.com/article/10.3390/molecules28083319/s1, Figure S1: XRD patterns of nanocomposites; Figure S2: Cyclic voltammograms for all nanocomposites (a) MoO_3_-(CNTs)_4%_ (b) MoO_3_-(CNTs)_4%_-PANI (c) MoO_3_-(CNTs)_(8%)_ (d) MoO_3_-(CNTs)_8%_-PANI nanocomposite; Figure S3: The charge/discharge curves of (a) MoO_3_-(CNT)_4%_ (b) MoO_3_-(MWCNT)_4%_-PANI (c) MoO_3_-MWCNT_S(8%)_ (d) MoO_3_-MWCNTs_(8%)_-PANI of all nanocomposites electrodes (e) Cycling performance of electrodes in the initial 10 cycles; Figure S4: The cyclic performance of (a) MoO_3_-(CNT)_4%_ (b) MoO_3_-(CNT)_4%_-PANI (c) MoO_3_-(CNT_S_)_8%_ (d) MoO_3_-(CNTs)_8%_-PANI nanocomposite nanocomposites at various current rates; Figure S5: The equivalent circuit and Nyquist plots of MoO_3_ nanocomposites; Table S1: Crystallite size of nanocomposites; Table S1: EIS fitting results of samples.
