# Supplementary material for: Flowers Like α-MoO3/CNTs/PANI Nanocomposites as Anode Materials for High-Performance Lithium Storage
Source: Molecules. 2023 Apr 8;28(8):3319. doi: 10.3390/molecules28083319 (PMC10143581; doi:10.3390/molecules28083319)
Supplement: Supplementary file 1 [file molecules-28-03319-s001.zip › molecules-2264285-supplementary.pdf]

**Supporting Information**

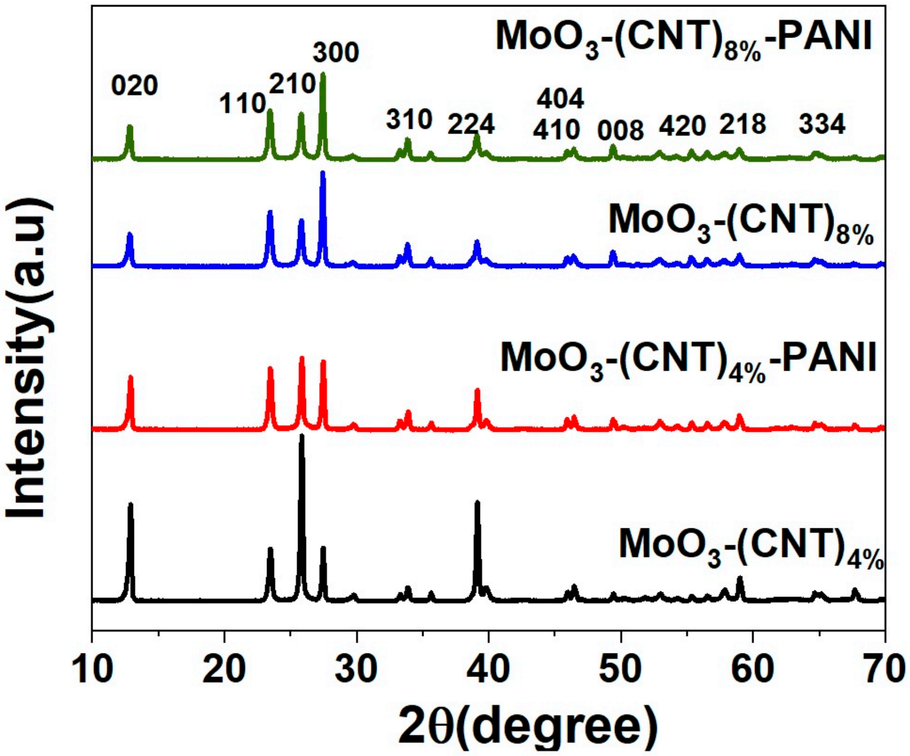


**Figure S1.** XRD patterns of nanocomposites.

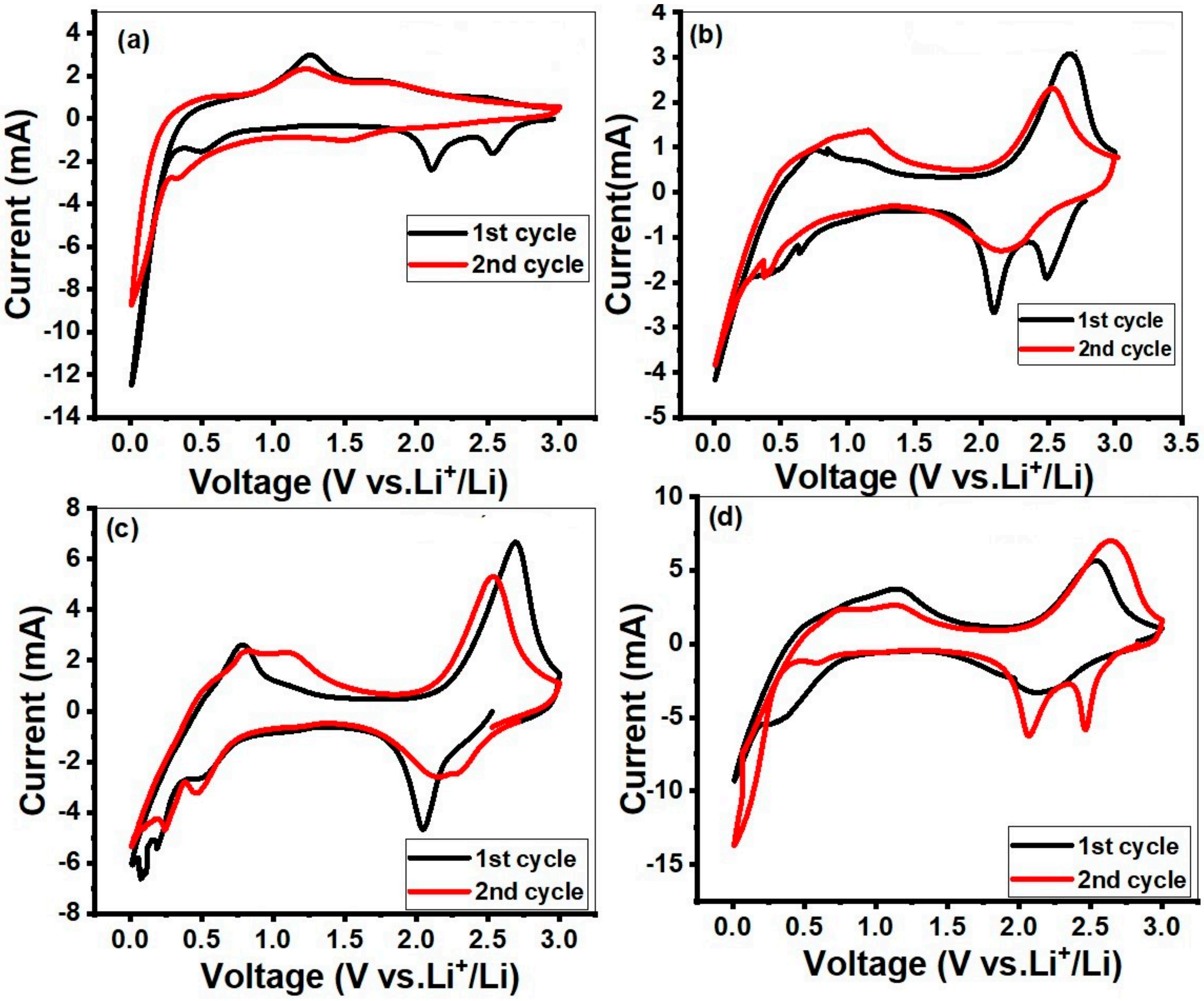


**Figure S2.** Cyclic voltammograms for all nanocomposites (a) $MoO_3$-$(CNTs)_{4\%}$ b) $MoO_3$-$(CNTs)_{4\%}$-PANI c) $MoO_3$–$(CNTs)_{(8\%)}$ c) $MoO_3$-$(CNTs)_{8\%}$-PANI nanocomposite.

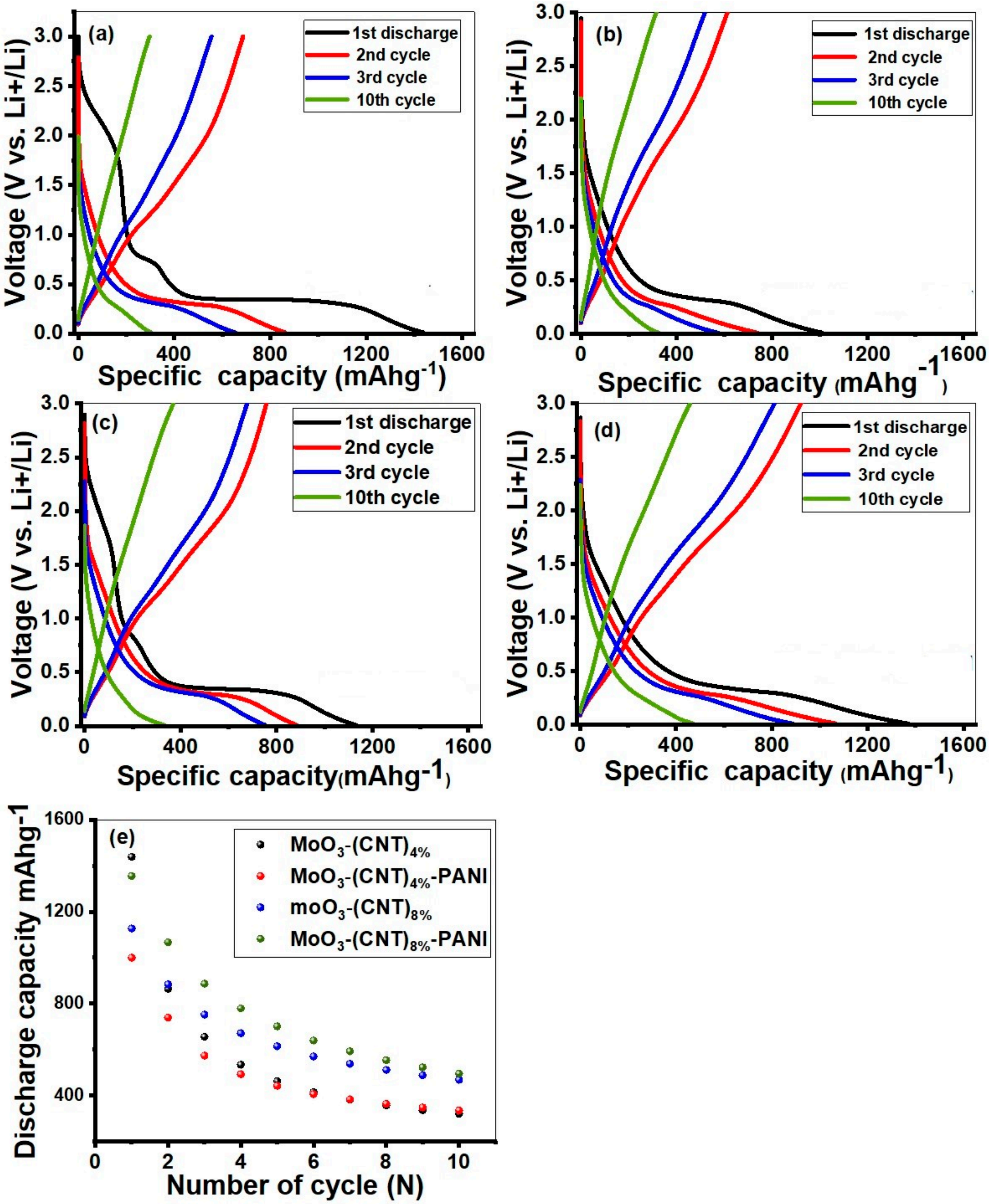
(a)
(b)
(c)
(d)
(e)
1st discharge
2nd cycle
3rd cycle
10th cycle
Voltage (V vs. Li+/Li)
Specific capacity (mAhg-1)
0.0
0.5
1.0
1.5
2.0
2.5
3.0
0
400
800
1200
1600
MoO3-(CNT)4%
MoO3-(CNT)4%-PANI
moO3-(CNT)8%
MoO3-(CNT)8%-PANI
Discharge capacity mAhg-1
Number of cycle (N)
2
4
6
8
10

**Figure S3.** The charge/ discharge curves of a) $MoO_3$-(CNT)4% b) $MoO_3$-(MWCNT)4%-PANI c) $MoO_3$–MWCNTs(8%) d) $MoO_3$-MWCNTs(8%)-PANI of all nanocomposites electrodes e) Cycling performance of electrodes in the initial 10 cycles.

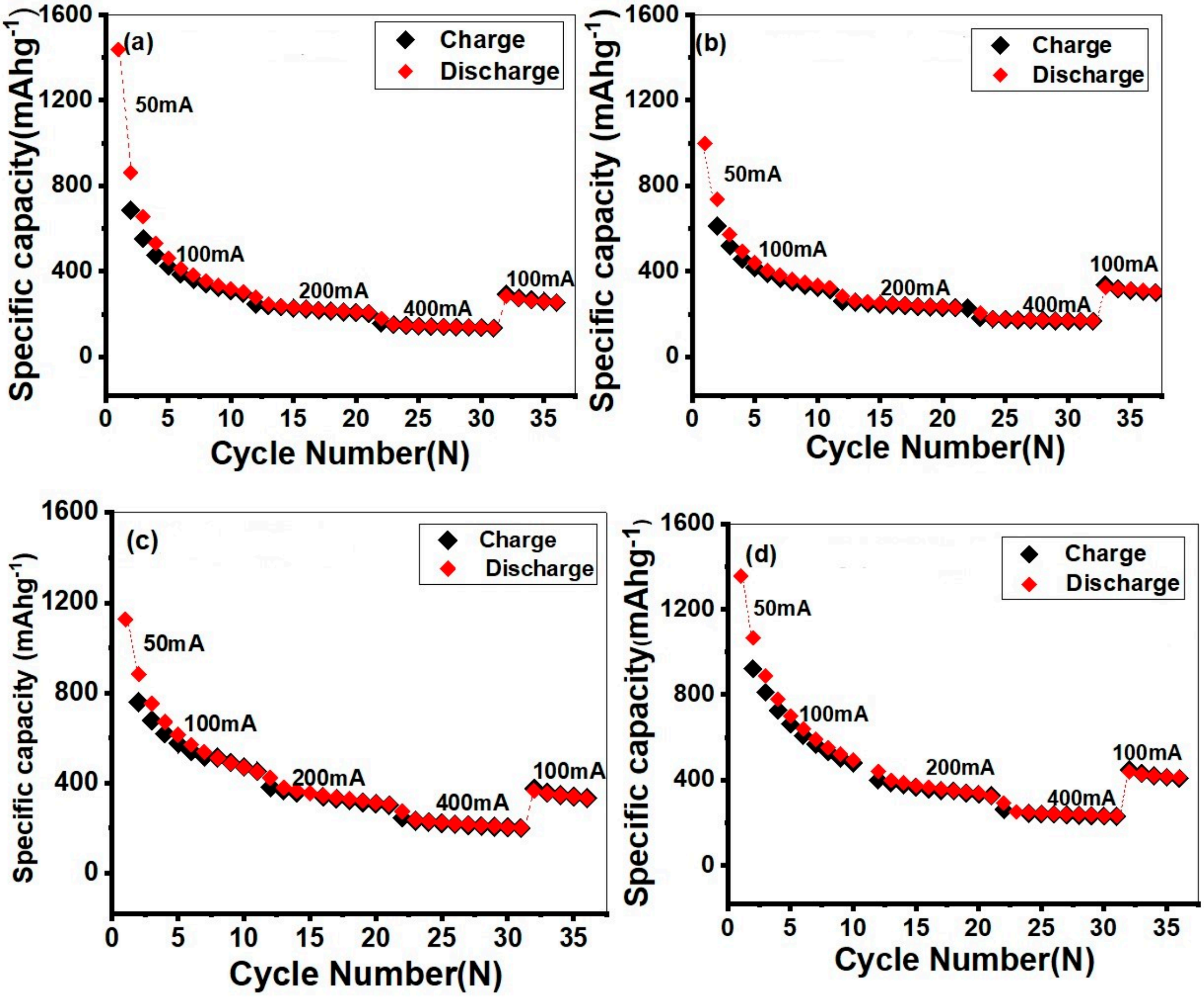


**Figure S4.** The cyclic performance of a) $MoO_3$-(CNT)4% b) $MoO_3$-(CNT)4%-PANI c) $MoO_3$–(CNTs)8% d) $MoO_3$-(CNTs)8%-PANI nanocomposite nanocomposites at various current rates.

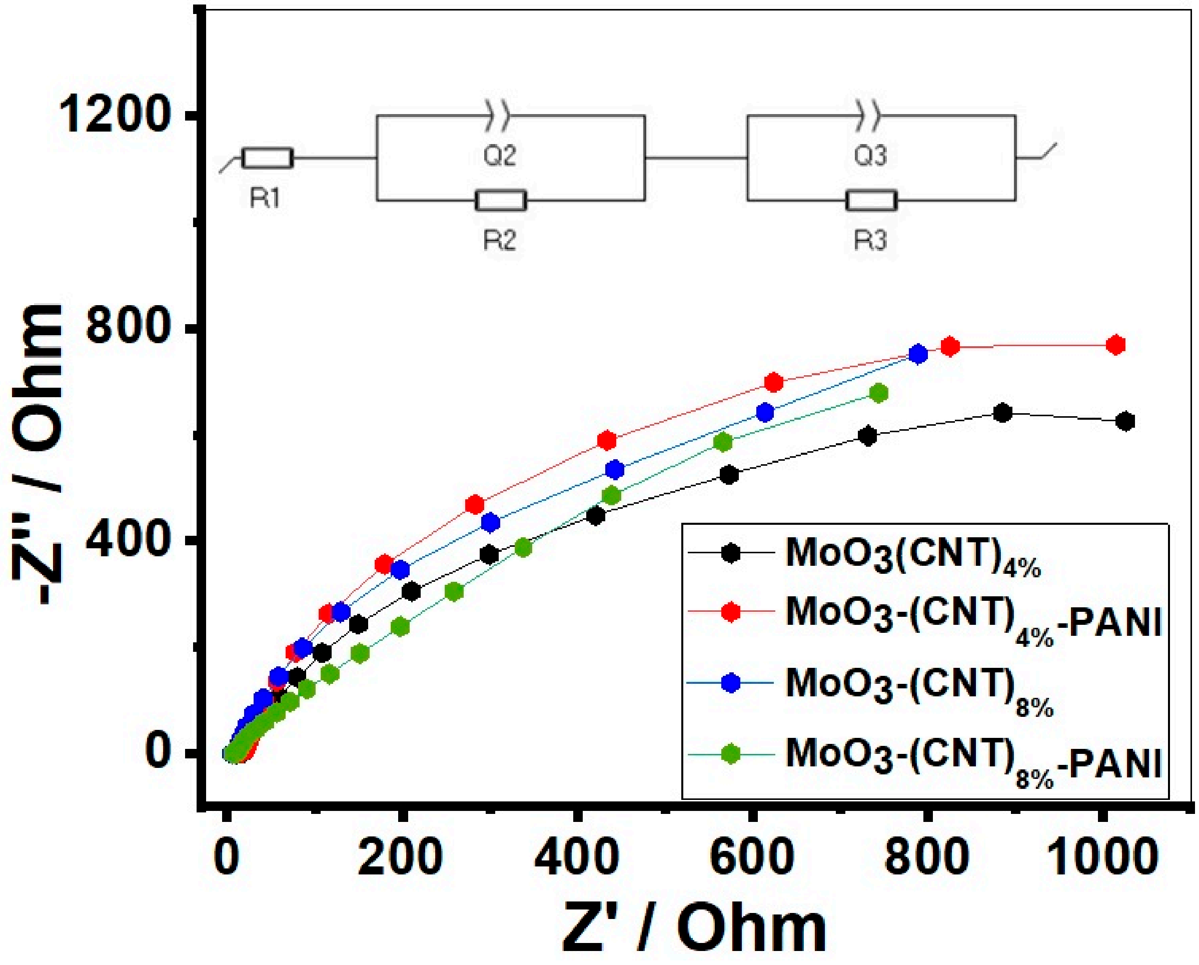


**Figure S5.** The equivalent circuit and Nyquist plots of $MoO_3$ nanocomposites.

**Table S1.** Crystallite size of nanocomposites

| **Samples** | **Crystallite size(nm)** |
|---|---|
| **$MoO_3$-(CNT)$_{4\%}$** | 48 |
| **$MoO_3$-(CNT)$_{8\%}$** | 46.5 |
| **$MoO_3$-(CNT)$_{4\%\text{-PANI}}$** | 48.8 |

| | |
|---|---|
| **$MoO_3$-(CNT)$_{8\%\text{-PANI}}$** | 37.6 |

**Table S2.** EIS fitting results of samples

| **Samples** | **$R_1$(Ω)** | **$R_2$(Ω)** | **$R_3$(Ω)** |
|---|---|---|---|
| **$MoO_3$-(CNT)$_{4\%}$** | 3.726 | 15.68 | 1007 |
| **$MoO_3$-(CNT)$_{8\%}$** | 4.072 | 9.923 | 739 |
| **$MoO_3$-(CNT)$_{4\%\text{-PANI}}$** | 3.674 | 14.77 | 871 |
| **$MoO_3$-(CNT)$_{8\%\text{-PANI}}$** | 5.279 | 13.92 | 783 |
